# Medical Opinion and Movement

**Published:** 1908-05-16

**Authors:** 


### Medical Opinion and Movement.

Although the results of the investigations of the Royal Commission on Tuberculosis are generally accepted as conclusive in favour of the transmission of tubercular infection from cattle to man, it is nevertheless instructive and interesting to obtain direct evidence of such transmission by concrete facts. Such evidence has recently been furnished by Dr. J. P. Watt in his annual report as medical officer of health for the county of Aberdeen. Two cases of tuberculous meningitis are reported as occurring in the family of a farm servant. The first patient, a girl aged nine, died with symptoms of tuberculous meningitis after an illness of about three weeks. A short time afterwards a second child, aged four years, was taken ill and died with similar symptoms. Owing to the possibility of the disease being cerebro-spinal meningitis, a post-mortem examination was made, and the original diagnosis was confirmed. A pure culture of tubercle bacilli was obtained from the cerebro-spinal fluid. The family was supplied with milk entirely from one cow which had been on the farm for a period of about four years. Examination of this cow disclosed a nodular mass on the udder, and the milk contained tubercle bacilli. The cow was killed, and a post-mortem examination showed extensive tuberculosis. These investigations were carried out by Dr. J. Adams, of the Pathological Department of the University, and by a comparison of the bacilli obtained from the child and from the cow he formed the opinion that they were both of the bovine type.

---

Even the most fundamental principles must be from time to time thoroughly probed, examined, and reconsidered in order that we may assure ourselves if they are formed upon a sound basis or require modification and adjustment. There is a very general conviction in the lay mind in regard to the weakening effect of lying in bed, and patients are often disposed to attribute the prostration of convalescence after an illness to their confinement in bed rather than to the disease from which they have suffered. On the other hand, the medical profession has in recent years perhaps rather intensified its affection for "rest in bed" as one of the first principles of treatment. After surgical operations, in particular, more or less prolonged rest has become the invariable custom, with only a gradual return to the normal activity of life. Recently, however, as was pointed out on pp. 621-2 of our issue for March 14, 1908, certain American and French surgeons have ventured to overthrow this custom, and have allowed their patients to get up at much shorter intervals after laparotomy operations, with apparently satisfactory results. Dr. George Chandler, who contributes further information concerning this method of after treatment, allows his patients to recover from the anæsthetic in a sitting posture, supported by a bed-rest. They are encouraged to sit up also on the following day, and on the third or fourth day they sit up on a chair, and on the fifth day they are allowed to walk about. His experience includes about 200 laparotomies, and he is thoroughly satisfied with the results. He finds that the upright posture inhibits nausea after the anæsthetic, promotes drainage to the pelvis, and prevents the weakness resulting from enforced rest.

---

It is always difficult to estimate the real intrinsic value of therapeutic methods involving more or less elaborate apparatus and the expenditure of considerable time and attention on the part of the patient and the medical attendant. All balneological forms of treatment come under this description, as well as electrical methods and the various forms of light treatment. While there are many enthusiasts among the profession for these different forms of treatment, there are not a few who from experience are led to the conclusion that much labour is involved with small result, and there is undoubtedly a very prevalent opinion that the psychic influence is an important factor in bringing about such favourable results as are sometimes reported. It is a notable fact that the enthusiastic pioneers of any such new form of treatment invariably obtain far better results than their subsequent imitators. As soon as a particular method loses the charm of novelty it also loses, at least to some extent, its charm to cure. An interesting discussion recently took place at Copenhagen on the treatment of cardiac affections by the Finsen light. Dr. Hasselbalch, Dr. Jacobæus, and others gave their experiences of the treatment, and claimed to have effected reductions in blood pressure, contractions of dilated hearts, and considerable relief from painful attacks of angina pectoris. These results were brought about by the prolonged action of the light rays upon the skin. Professor Israel Rosenthal, however, expressed surprise at the roseate tints in which the results of the treatment were depicted, and incredulity was also displayed at the meeting by the suggestion of the potency of the psychic factor. That any lasting beneficial effect can be obtained on a diseased heart by the action of light rays on the skin appears *a priori* somewhat improbable.

---

Continental physicians are undoubtedly more enterprising in their clinical methods of investigation and examination than ourselves. Gastric lavage was adopted as a routine method both for examination and treatment of stomach conditions some time before it received recognition in this country. One of the latest methods for investigating the functions of the stomach is known as the desmoid reaction. It is based upon the fact that connective tissue is digested by the normal gastric juice, but not by intestinal secretions. In order to test the digestive powers of the stomach, therefore, a tablet of methylene blue is enclosed in a membrane of caoutchouc tied with a catgut ligature, and this is administered to the patient immediately after the midday meal. The catgut ligature is digested by the gastric juice in a certain interval of time, varying according to the conditions of gastric digestion, and the methylene blue thus liberated appears in the urine. By observing the time at which the methylene blue first appears in the urine it is claimed that conclusions can be drawn as to the functional digestive powers of the stomach. If the reaction is retarded, either a deficiency of gastric secretion or failure of proper evacuation is indicated. When the reaction occurs only after some days pyloric stenosis is supposed to be indicated. The reaction appears to be a useful aid in many cases to clinical diagnosis, although it is, like most of these reactions, liable to fallacies. A late appearance of the methylene blue in the urine may, for instance, depend upon a renal or circulatory insufficiency. A detailed account of the method is given by MM. Dubourdieu and Lemaire in the March issue of the *Bulletin des Sciences Pharmacologiques*.

DR. HEARSAY, Principal Medical Officer Nyassaland, quoting from a report of the Principal Medical Officer of Uganda and East Africa, brings forward some interesting facts in the treatment of sleeping sickness by atoxyl. He states that there appears to be no doubt that more than half the cases of sleeping sickness show marked temporary improvement in their general bodily health after atoxyl treatment, and the enlarged glands diminish in size in nearly every case. It has, however, been noted that even when cases have been taken early in hand about 34 per cent. die by the end of the year. The following symptoms suggestive of atoxyl intoxication have been observed—giddiness, colic and diarrhœa, dimness of vision and other visual disturbances; paresis of lower limbs with increased knee-jerks, and sudden death; 20 per cent. of Kopke's cases had vision affected, and half of these became totally blind; in the segregation camp at Busiro, Uganda, out of 571 cases, 6.5 per cent. had their vision affected, and 2 per cent. became totally blind, the visual affection not appearing to bear any relation to the amount of atoxyl given. The consensus of opinion among medical officers in Uganda is, however, that, provided the quality of atoxyl is above suspicion, the good results outweigh any bad results that may possibly occur, and that, until a better substance can be found, there is justification for going on with the treatment.

BRUDZINSKI, in a communication entitled "Contra-lateral reflexes of the lower limbs," claims to have discovered that in certain nervous affections and neuropathic conditions of children, particular forms of associated movements are present. Thus, if the sound limb of a child suffering from hemiplegia be forcibly flexed, the paralysed limb will be seen to execute a movement of extension which has all the characters of a reflex. The contrary phenomenon is not produced, however. He calls this movement the reciprocal contra-lateral reflex. On the other hand, in some other cases a second type of reflex—the identical contra-lateral—is found. In this type forced flexion of the sound limb produces a movement of flexion in the affected limb. Again, forced extension may produce a movement of extension in other cases. None of these reflexes are to be found in normal children, and the results were also negative in two cases of chorea and one of tetanus examined. The "identical" reflex is said to be constant in cases of tuberculous and cerebro-spinal meningitis. The "reciprocal" reflex is the more common in hemiplegia. In "backward" children (mongols, idiots, etc.), and in children suffering from Little's disease, one or other reflex has been found. The utility of the discovery consists not only in the presence of the reflexes being diagnostic of disease, but in their absence or diminution being prognostic of recovery, since they tend to disappear as the child gets well. Brudzinski insists on the examinations being made more than once, since it is easy to mistake a child's voluntary movements for reflexes.

MAYNARD LADD in the *Archives of Pediatrics* raises a plea for greater accuracy and uniformity in prescribing starch in infant feeding. He refers especially to cereal decoctions, of which barley-water is, of course, the most popular, and he deplores the lack of uniformity of method in the preparation of these diluents, which, in his opinion, partly explains the present divergence of medical opinion as to an infant's ability to digest starch, and as to the advisability of giving farinaceous food in the early months of life. An analysis of six decoctions of barley, prepared according to the formulæ of six different writers, showed a variation of from 0.64 to 6.00 per cent. of starch. He proposes a 3.50 per cent. decoction of starch as a standard. This uniform stock solution he has prepared by using $2\frac{1}{3}$ ounces of prepared barley (Robinson's) to a quart of water, cocking for thirty minutes, and adding sufficient water to make one full quart. The author hopes that it will become possible in the infant's dietary of the future for starch to be prescribed in percentages, just as fats, sugars, and proteids have been prescribed. He says "it is of unquestioned advantage to think and write in percentages, for in this way only may we express scientifically the manner in which the problems of infant feeding are to be worked out so that the experience of one man will be of value to another."

IN 1890 Demars and Marx published an account of a new operation for the relief of chronic obesity. The operation consisted in the removal of as large an amount as possible of the supra-aponeurotic abdominal fat. Two transverse incisions were made, one just below the umbilicus and the other above the pubis, the incisions curving round to meet at the sides, and so isolating a large oval piece of the abdominal skin which, together with the subcutaneous tissues down to the aponeurosis of the recti, was completely removed. The patient did well. Schulz has recently operated on two cases of obesity, employing a similar method. In one case the piece of fat and skin removed weighed close upon 5 kilogram, in the other 4.2 kilogram. The enormous wound was closed with interrupted sutures, and a drain was inserted in each case. Both the patients were bad subjects for operation—the one, a woman of 30, suffering from a chronic affection of the kidneys; and the other, a woman aged 53 years, and a chronic sufferer from cholelithiasis: but in each case recovery was uneventful. One case was operated upon five years ago, and the other six months later, and since then there has been comparatively little deposit of fat below the scar. The author speaks very highly of the operation, but admits that it should be employed in selected cases only.

---

THE German Surgical Congress, which concluded its annual session at Berlin on April 26 last, demonstrated very well the advances made during the last few years in several departments hitherto considered the borderland between medicine and surgery. Professor Trendelenburg, for instance, described a new operation for pulmonary embolism, which consists essentially in opening the right ventricle and removing the clot from the orifice of the pulmonary artery. The operation, which has, of course, to be done " against time," was successfully performed on a calf, and its possibilities for the human patient have yet to be determined. Cardiac surgery has of late years been enormously furthered by the brilliant and heroic undertakings of Professors Rehn, Gluck, and Tuffier; but hitherto such things as organic disease of the heart and cardiac embolism have been considered outside the domain of practical operative surgery. Surgical interference has been frequently proposed, and in England Sir Lauder Brunton has theoretically defended such interference in extreme cases of mitral stenosis. But though the heart allows of a comparatively large amount of handling and pressure, the other effects of such operations are not within the power of the surgeon to control. Nevertheless, the fact that Professor Trendelenburg's new operation has been successfully accomplished on animals, and that it has been actively and earnestly discussed, should make it an operation which in certain cases it would be justifiable to attempt.

---

THE other papers read before the Congress call for less detailed comment. None of them presented what was outstandingly novel or original, with the brilliant exception of Dr. Förster of Breslau's demonstration at the meeting of the orthopædic section. This worker showed two cases of spastic paraplegia in infants, in which marked improvement had followed the original operation of complete section of the posterior roots in the lower dorsal and lumbar region. About the merits of the operation it is far too early in the day to argue; more observations are wanted, and the two cases demonstrated need further watching before we are in a position to agree that in each a real " cure " has been effected. That, however, by no means detracts from the honour due to Dr. Förster for devising and perfecting a method which, after the demonstration of the excellent results obtained in his two cases, is worthy the careful consideration of surgeons who have to deal with this condition. The physiological factors which have led to the cure claimed in the two demonstrated cases are not easy to point out, and for that reason probably, the operation was not discussed at length by the Congress. The principle of the operation is, we believe, quite new, and the results obtained are excellent.

EXOPHTHALMIC goitre, from the point of view of diagnosis and treatment, is dealt with in an interesting communication by Jackson and Mead to the *Boston Medical and Surgical Journal.* In the diagnosis of this condition the authors lay stress on the significance of a vascular murmur over the enlarged thyroid gland. In 87 per cent. of their cases such a murmur was present. Apart from the cardinal symptoms—goitre, exophthalmos, tachycardia, and tremor—the authors advise careful search for evidences of Graves' disease in the nervous, digestive, and cutaneous systems. Emaciation and dyspnœa they regard as particularly important symptoms in doubtful cases. With reference to the variations in degree and type that are seen in Graves' disease, they believe that this condition, whether it be called complete or incomplete, primary or symptomatic, is really one and the same disease. In the treatment of exophthalmic goitre the authors claim to have achieved great success from the prolonged administration of the neutral hydrobromide of quinine. Their method is to give 5 grains of this salt in cachets three times a day. Indications of cinchonism, which are less frequent than with other preparations of quinine, call for a reduction in the daily quantity. The authors insist upon the necessity for perseverance in this method of treatment. A month's trial is usually not enough to produce any obvious improvement, while as much as 18 months or two years may be necessary for the establishment of a complete cure. A possible objection to the continuous administration of this substance for long periods of time is that it is more than twice as expensive as potassium iodide. Apart from this, the treatment seems simple and worthy of a thorough trial.

---

THE cutaneous manifestations observed in rheumatism in children form the subject of a paper by Dr. J. F. Schamberg, of Philadelphia, in a recent number of the *Monthly Cyclopædia of Practical Medicine.* As the author remarks, there is no skin lesion constantly or characterisically associated with rheumatic fever. Rheumatic nodules are not cuticular, but sub-cuticular. The commonest eruptions are exudative erythema multiforme (usually gyrate or papular in distribution), erythema nodosum, erythema scarlatiniforme, urticaria, and various forms of purpura. These all belong to what has been called the " erythema group of skin diseases," between the various members of which there is a close causal relationship. The author shares the views expressed by Harrison on the subject of erythema nodosum: in spite of its frequent association with rheumatic fever, he doubts whether this is a genuine rheumatic process. In the vast majority of cases, according to him, erythema nodosum occurs independently of any other active systemic disease. He regards it as a toxic affection, which may be produced by a variety of poisons. The rheumatic infection merely constitutes one of the most frequent poisons capable of producing the disease. In the same way the author doubts whether purpura rheumatica also is a true and essential rheumatic process.

